# Three‐dimensional reconstruction of the knee joint based on automated 1.5T magnetic resonance image segmentation: A feasibility study

**DOI:** 10.1002/jeo2.70361

**Published:** 2025-07-18

**Authors:** Charles Pioger, Laura Marin, Yvon Gautier, Julien Cléchet, Pierre Imbert, Christian Lutz, Étienne Cavaignac, Bertrand Sonnery‐Cottet

**Affiliations:** ^1^ Department of Orthopaedic Surgery Ambroise Paré Hospital Boulogne‐Billancourt France; ^2^ Laboratory AMIS, UMR 5288 CNRS Paul Sabatier University Toulouse France; ^3^ AREAS Grenoble France; ^4^ Centre Orthopédique Santy Lyon France; ^5^ Clinique Les Lauriers, ELSAN Fréjus France; ^6^ Institut de Chirurgie Orthopédique et Sportive à Strasbourg Strasbourg France; ^7^ Department of Orthopaedic Surgery Pierre Paul Riquet Hospital, CHU de Toulouse Toulouse France

**Keywords:** 3D knee reconstruction, deep learning, magnetic resonance imaging, preoperative planning, segmentation, surface laser scanning

## Abstract

**Purpose:**

To validate the accuracy of three‐dimensional (3D) bone and cartilage reconstructions of the distal femur and proximal tibia derived from 1.5 Tesla magnetic resonance imaging (MRI), using fully automated and semi‐automated segmentation methods, compared to surface laser scanning (LS) as the reference standard.

**Methods:**

Eleven fresh‐frozen cadaveric knees were imaged using a 1.5 T MRI scanner. Manual (MS), fully automated (A), and semi‐automated (SA) segmentations were performed to generate 3D models of the distal femur and proximal tibia. A transformer‐based deep learning model (UNet‐R) was used for automated segmentation. Laser surface scanning provided high‐resolution ground‐truth 3D models. Point‐to‐surface distances between MRI‐based and LS‐derived models were calculated to assess reconstruction accuracy. Bland‐Altman analyses were performed to compare segmentation methods. Time to generate 3D models was recorded for each method.

**Results:**

The mean absolute point‐to‐surface distance for femoral models was 1.19 mm (±0.42) for MRI A, 1.05 mm (±0.09) for MRI SA, and 0.99 mm (±0.08) for MRI MS. For tibial models, the corresponding values were 1.54 mm (±1.02), 1.03 mm (±0.17), and 0.93 mm (±0.14), respectively. MRI A showed larger variability, which required manual correction. Time analysis revealed significant efficiency gains: 27 s for MRI A, 1520 s for MRI SA, and 14,191 s for MRI MS (*p* < 0.001). Bland‐Altman plots confirmed improved agreement of MRI SA with MRI MS.

**Conclusions:**

MRI‐based 3D reconstructions of the knee using a 1.5 T system and semi‐automated segmentation achieved sub‐millimetre accuracy comparable to manual segmentation and significantly outperformed fully automated models in precision, while substantially reducing segmentation time. These findings support the integration of AI‐assisted 3D reconstruction into preoperative planning workflows for knee ligament surgery, offering a reliable, radiation‐free alternative to CT‐based modelling.

**Level of Evidence:**

Level IV, controlled laboratory study.

**Abbreviations**
1.5T: 1.5 Tesla
A: automated segmentation
ACL: anterior cruciate ligament
AI: artificial intelligence
CNN: convolutional neural network
CPD: coherent point drift
CT: computed tomography
DICOM: Digital Imaging and Communications in Medicine
FOV: field of view
GRAPPA: GeneRalized Autocalibrating Partial Parallel Acquisition
IQR: interquartile range
LOA: limits of agreement
LS: laser surface (scanning)
MC: manual correction
MRI: magnetic resonance imaging
MS: manual segmentation
PSI: personalised surgical instrument
RMS: root mean square
SA: semi‐automated segmentation
SD: standard deviation
STL: standard tessellation language (format)
TE: echo time
TR: repetition time
UNet‐R: transformer‐based deep learning segmentation architecture
W&B: Weights & Biases (outil de suivi d'entraînement IA)

## INTRODUCTION

Knee ligament injuries are frequent traumatic events, with diagnosis based on a combination of clinical symptoms, comprehensive physical examination, and imaging [9, 16]. Although conventional radiography provides limited diagnostic value, magnetic resonance imaging (MRI) remains the gold standard, as it not only confirms ligament ruptures but also identifies associated meniscal and cartilage injuries [15].

The increasing integration of three‐dimensional (3D) joint modelling in orthopaedic surgery has significantly enhanced surgical planning and intraoperative guidance [19]. However, current 3D model generation predominantly relies on computer‐assisted navigation or computed tomography (CT). The latter is not routinely performed in the preoperative workup of ACL injuries for example, due to its ionising radiation exposure [1] and limited sensitivity to visualise other anatomical structures of interest such as menisci, ligaments, but also for cartilage tissue.In this context, MRI‐based 3D knee modelling emerges as a promising alternative, minimising costs and eliminating radiation exposure. However, current MRI‐based 3D reconstruction methods rely primarily on manual segmentation, which remains labour‐intensive and time‐consuming. Recent advancements in deep learning, a subset of machine learning, have revolutionised automated segmentation of anatomical structures from medical imaging, enabling faster and more reproducible 3D reconstructions [7, 11, 21]. To date, most studies validating MRI‐based 3D bone models have been conducted using 3‐Tesla (3 T) MRI [3, 4, 28] and typically compared them to standard CT scans [17, 23]. Therefore, validating MRI‐derived bone and cartilage models at 1.5 T against surface laser scanning (ground‐truth surface) represents a crucial step in assessing whether 1.5 T MRI can achieve equivalent precision in generating high‐fidelity 3D bone models [12, 13]. Such validation could support its adoption into routine preoperative workflows, particularly for knee ligament surgery; or provide additional information for the manufacture of Personalised Surgical Instrument (PSI) based on cartilage tissue [29].

This study aims to validate the accuracy of semi‐automated and fully automated segmentation‐based 3D bone and cartilage reconstructions of the distal femur and proximal tibia derived from 1.5 T MRI, by comparing them to surface laser scans (LS). We hypothesise that the integration of an automatic segmentation algorithm in the workflow for generating a 3D model from an MRI scan greatly speed up 3D model generation, while achieving precision equivalent to that of surface laser scanning.

## METHODS

### Ethical considerations

Institutional review board approval (COS‐RGDS‐2022‐12‐025) was granted for this study which was conducted in strict accordance with the institutional ethical standards governing research involving human participants.

### Study design

A total of 15 fresh‐frozen cadaveric knees without a history of prior knee surgery were included in this study. The mean age of the donors was 60.5 years (range, 33–88); six were female, and nine were male. The sample size was determined based on prior cadaveric studies assessing the feasibility of 3D bone modelling derived from MRI [13, 17, 23]. Specimens were thawed at room temperature for over 24 hours before imaging and dissection. Knees presenting with osteoarthritis or pathological laxity were excluded before dissection. A radiographic assessment followed by arthroscopic examination was performed to exclude osteoarthritis and verify the integrity of the anterior cruciate ligament (ACL).

### MRI acquisition and image processing

Each specimen underwent MRI scanning using a MAGNETOM Sola, 1.5 Tesla (Siemens Healthcare, Erlangen, Germany). The MRI protocol included standard pulse sequences along with a 3D dual‐echo gradient imaging with echo times (TE) of 65 ms. Additional acquisition parameters were as follows: repetition time (TR) = 900 ms, flip angle = 9°, 192 axial slices, slice thickness = 0.9 mm, field of view (FOV) = 200 mm × 288 mm, phase oversampling: 30%, slice oversampling: 9%, acquisition matrix = 320 × 320, parallel imaging factor (GRAPPA) = 2, voxel size = 0.9 mm × 0.9 mm × 0.9 mm, bandwidth = 325 Hz/px, turbo factor: 40.

Following acquisition, three distinct image processing methods were applied:

1. Manual Segmentation group (MRI MS): MRI images (DICOM format) were manually segmented by an experienced operator (CP), familiar with manual MRI knee segmentation using ITK‐SNAP 3.0 software (Cognitica, Philadelphia, PA, USA). Manual segmentation was performed based on anatomical landmarks including bone and cartilage. Each segmentation was validated by one independent senior knee surgeon and a musculoskeletal radiologist, with discrepancies resolved by consensus among all two evaluators. The resulting 3D models were exported in standard tessellation language (.stl) format.
2. Automated Segmentation group (MRI A): A deep learning algorithm (AREAS, Grenoble, France) was employed for automatic segmentation. The architecture and training details of this artificial intelligence (AI) model are described below.
3. Semi‐Automated Segmentation group (MRI SA): Each 3D model from the automatic segmentation group was examined on ITK snap by a senior knee surgeon and a musculoskeletal radiologist. In the event of anatomical aberration, the model was manually corrected by an experienced operator before being reintegrated into the semi‐automated group. The time required to generate the 3D models was recorded for each image processing group (MS, A, and SA).

### Deep learning algorithm for automated segmentation

A transformer‐based deep‐learning model, UNet‐R [5], was developed and trained for the automatic segmentation of the knee anatomy. The input MRIs consisted of 3D proton density fat‐saturated scans, acquired along sagittal axis with a size of 512 × 512 × 160. The model outputs a corresponding 3D segmentation map covering 12 anatomical structures: bones (femur, tibia, patella, and fibula), cartilages (femoral and tibial), menisci (internal and external), and ligaments (cruciate and lateral).

A proprietary data set of anonymized knee MRIs was collected under institutional ethical approval (COS‐RGDS‐2022‐12‐025). The data set was randomly divided into training and validation subsets. Preprocessing steps included intensity normalisation and the application of several data augmentation techniques to improve model robustness. Specifically, spatial augmentations were used: foreground extraction to isolate relevant anatomical structures, random axis flips (x, y and z), and 90° rotations. These operations aimed to increase spatial variability and enhanxce the model's ability to generalise across different anatomical presentations.

Training was conducted using PyTorch and Monai frameworks, on an NVIDIA RTX 4080 Ti GPU. The model was optimised using the Adam optimiser (learning rate = 1e^−5^, weight decay = 1e^−6^), a batch size of 1, a patch batch size of 4 (i.e., the number of extracted patches per MRI), and trained for 6000 epochs. The Dice coefficient was used for performance evaluation across all structures. Training lasted approximately 36 h, and inference required approximately 22 s per MRI. Weights & Biases (W&B) was used for experiment tracking, as well as data and model versioning.

#### Dissection and surface laser scanning

After imaging, all soft tissues were meticulously removed, preserving only the bony and cartilaginous structures of the distal femur and proximal tibia. The specimens were then subjected to 3D surface laser scanning (scanner (ATOS Q 12 M, Carl Zeiss GOM Metrology GmbH, Schmitzstraße 2, 38122 Braunschweig, Germany) to generate ground‐truth surface models with a precision of 30 μm. A 360° scan was performed with a field of view of 120 mm by 90 mm, and a resolution of 0.03–0.12 mm point distance. All 3D models obtained with this surface laser scanner were exported in.stl format and used as the ground truth for comparison with the 3D models derived from MRI in automatic, semi‐automatic, and manual methods.

### Comparison of MRI‐based versus laser‐scanned (LS) 3D reconstructions

**Figure 1:**
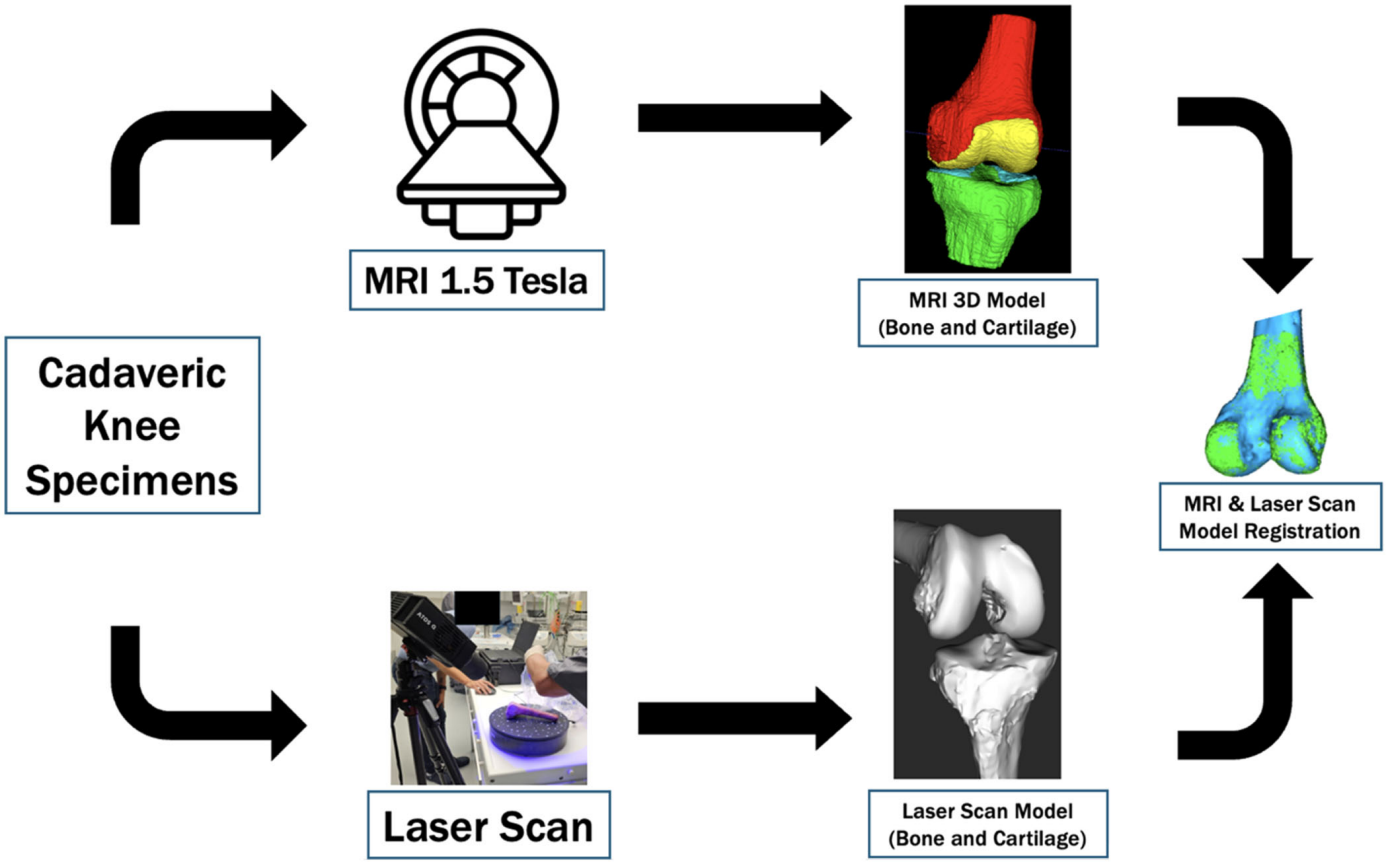
Comprehensive outline of the study's image acquisition and processing pipeline. MRI, magnetic resonance imaging.

The MRI‐based 3D bone models (MS, A and SA) and laser scanning models of cadaveric specimens were compared by analysing the distribution of absolute surface‐to‐surface distances between each pair of superimposed models, including MRI MS versus LS, MRI A versus LS, and MRI SA versus LS (Figure 1). This superimposition was performed using a three‐dimensional (3D‐3D) registration algorithm.

### Precision evaluation

The minimum point‐to‐surface distances were calculated using a dedicated algorithm. A Coherent Point Drift (CPD) registration algorithm was employed to align the predicted 3D models, whether generated automatically (MRI A), manually (MRI MS), or after correction (MRI SA), with the surface scanner data for the tibia and femur bone structures.

The precision of the predicted 3D model was evaluated by calculating the absolute point‐to‐surface distance between each point on the surface scanner data and its closest corresponding point on the registered predicted 3D structure. This point‐wise evaluation provided a quantitative measure of the model's accuracy. The computation allowed for the assessment of regional discrepancies between models while also providing an absolute measurement of surface distances.

A positive signed distance indicated that the analysed model was larger than the reference model in a given region, whereas a negative signed distance revealed that the analysed model was smaller. For LS versus MRI comparisons, the reference models derived from LS were considered the gold standard (ground truth), ensuring a precise regional evaluation of geometric variations between models while validating the ability of each modality to accurately replicate bone geometry.

### Statistical analysis

All statistical analyses were performed using IBM SPSS Statistics (v. 24, IBM, Armonk, NY, USA). Data normality was assessed using the Shapiro–Wilk test. Normally distributed variables were expressed as mean ± standard deviation (SD), while non‐normally distributed variables were presented as median with interquartile range (IQR). The agreement between segmentation methods was evaluated using Bland–Altman plots, comparing manual segmentation (MRI MS), semi‐automated segmentation with manual correction (MRI SA), and fully automated segmentation (MRI A) against laser scan (LS) reference models. The analysis focused on the distribution of absolute point‐to‐surface distance differences, allowing for the identification of systematic biases and variability. Limits of agreement (LOA) were calculated as mean difference ± 2 SD. Paired comparisons between segmentation methods were conducted using paired t‐tests for normally distributed data and Wilcoxon signed‐rank tests for non‐parametric distributions. Additionally, outlier analysis was performed to examine the influence of extreme values on segmentation variability. Given the presence of outliers in certain specimens, sensitivity analyses were conducted to evaluate their impact on the mean point‐to‐surface differences and LOA estimates. Statistical significance was set at *p* < 0.05 for all comparisons.

## RESULTS

### Specimen selection and segmentation performance

Among the 15 cadaveric knee specimens initially included, four were excluded due to advanced osteoarthritis or ACL deficiency. As a result, 11 knees were analysed. Although a notable proportion of specimens were excluded, the size and quality of the final sample remain comparable or superior to those reported in previous studies [12, 13].

Three of the AI‐generated 3D knee models (Specimens 5, 8 and 9) contained segmentation outliers and required manual correction, indicating the need for refinement in fully automated segmentation approaches.

The process times according to the treatment modalities were very different with an average time of 3.9 h for manual segmentation, 27 s for automated segmentation and 25 min for semi‐automatic segmentation.

### Point‐to‐surface distance analysis

#### Femoral side

For the MRI A versus LS comparison, the mean absolute point‐to‐surface distance was 1.19 mm (SD 0.42 mm), while MRI SA versu LS yielded a lower mean difference of 1.05 mm (SD 0.09 mm) (Table 1). The MRI MS versus LS comparison demonstrated the closest correspondence, with a mean difference of 0.99 mm (SD 0.08 mm). Across individual specimens, MRI A exhibited the highest variability, particularly in Specimen 5, where the maximum discrepancy reached 2.44 mm, whereas MRI SA and MRI MS showed consistently lower deviations.

**Table 1:** The average (SD) absolute point to surface distance of the 3D distal femoral bone models between the LS versus MRI A, SA and MS.

Specimens femur	MRI A vs. LS absolute in mm (min–max)	MRI SA with MC vs. LS absolute in mm (min‐max)	MRI MS vs. LS absolute in mm (min‐max)
1	1.13 (0.01–14.9)	1.13 (0.01–14.9)	1.0 (0.01–15.0)
2	1.26 (0.02–17.2)	1.26 (0.02–17.2)	0.94 (0.02–13.0)
3	1.0 (0.03–12.0)	1.0 (0.03–12.0)	1.13 (0.04–16.6)
4	0.96 (0.05–11.6)	0.96 (0.05–11.6)	0.89 (0.02–12.3)
5	2.44 (0.03–20.9)	1.02 (0.04–14.2)	0.89 (0.02–11.8)
6	1.06 (0.02–15.2)	1.06 (0.02–15.2)	1.02 (0.04–14.16)
7	1.0 (0.02–14.5)	1.0 (0.02–14.5)	0.97 (0.04–15.26)
8	1.14 (0.04–13.4)	1.04 (0.03–13.0)	1.03 (0.03–12.4)
9	0.98 (0.02–14.6)	0.99 (0.02–14.7)	0.98 (0.03–15.0)
10	0.98 (0.03–12.5)	0.98 (0.03–12.5)	0.98 (0.03–12.6)
11	1.13 (0.03–14.1)	1.13 (0.03–14.1)	1.13 (0.02–14.5)
Average (SD)	1.19 (0.42)	1.05 (0.09)	0.99 (0.08)

Abbreviations: A, automated; LS, laser surface; MC, manual correction; MRI, magnetic resonance imaging; MS, manual segmentation; SA, semi‐automated; SD, standard deviation.

#### Tibial side

For the tibial segmentations, MRI A versus LS had a mean absolute difference of 1.54 mm (SD 1.02 mm), while MRI SA versus LS demonstrated improved concordance with a mean difference of 1.03 mm (SD 0.17 mm) (Table 2). The MRI MS vs. LS comparison exhibited the strongest consistency, with a mean difference of 0.93 mm (SD 0.14 mm). The greatest deviation was noted in Specimen 5, where MRI A showed the highest discrepancy (4.27 mm, range 0.06–19.7 mm).

**Table 2:** The average (SD) absolute point to surface distance of the 3D proximal tibial bone models between the LS versus MRI A, SA and MS.

Specimens tibia	MRI A vs. LS absolute in mm (min–max)	MRI SA with MC vs. LS absolute in mm (min–max)	MRI MS vs. LS absolute in mm (min–max)
1	1.05 (0.02–11.9)	1.05 (0.02–11.9)	0.94 (0.04–6.9)
2	1.22 (0.04–15.9)	1.22 (0.04–15.9)	0.89 (0.02–4.8)
3	1.31 (0.02–12.6)	1.31 (0.02–12.6)	0.96 (0.01–15.2)
4	1.06 (0.03–8.7)	1.06 (0.03–8.7)	1.13 (0.03–11.8)
5	4.27 (0.06–19.7)	1.01 (0.02–12.6)	0.96 (0.02–8.8)
6	1.05 (0.02–15.6)	1.05 (0.02–15.6)	0.98 (0.04–12.8)
7	0.93 (0.02–5.9)	0.93 (0.02–5.9)	0.74 (0.04–3.6)
8	2.01 (0.07–22.7)	0.8 (0.03–4.4)	0.8 (0.03–4.0)
9	2.28 (0.07–21.7)	1.14 (0.02–13.0)	1.15 (0.03–13.1)
10	0.75 (0.02–3.7)	0.75 (0.02–3.7)	0.72 (0.02–3.6)
11	0.98 (0.02–4.5)	0.98 (0.02‐4.5)	0.99 (0.03–4.7)
Average (SD)	1.54 (1.02)	1.03 (0.17)	0.93 (0.14)

Abbreviations: A, automated; LS, laser surface; MC, manual correction; MRI, magnetic resonance imaging; MS, manual segmentation; SA, semi‐automated; SD, standard deviation.

### Bland–Altman agreement analysis

#### Femoral side

**Figure 2:**
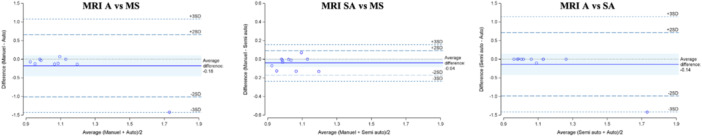
Bland–Altman plots comparing MRI‐based three‐dimensional (3D) femoral reconstructions using different segmentation methods. A, automated; MRI, magnetic resonance imaging; MS, manual segmentation; SA, semi‐automated.

The average difference between MRI A and MRI MS was 0.18 mm with limits of agreement (LOA) ranging from −0.36 mm to +0.76 mm (Figure 2). The highest discrepancy was observed in specimen 5, where MRI A showed the greatest variability compared to MRI MS, with a difference reaching 1.49 mm.

The MRI SA versus MRI MS comparison demonstrated a mean difference of 0.04 mm with LOA from −0.12 mm to +0.24 mm. The agreement was more consistent compared to MRI A, suggesting that manual correction significantly improved segmentation accuracy.

The average difference between MRI SA and MRI A was 0.14 mm, with LOA from −0.22 mm to +0.50 mm. The agreement showed that MRI SA tended to correct some of the discrepancies observed in MRI A.

#### Tibial side

**Figure 3:**
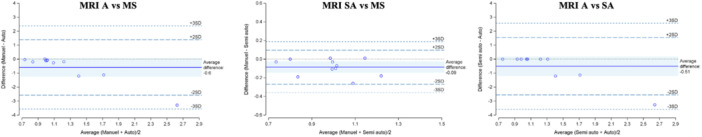
Bland‐Altman plots comparing MRI‐based three‐dimensional (3D) tibial reconstructions using different segmentation methods. A, automated; MRI, magnetic resonance imaging; MS, manual segmentation; SA, semi‐automated.

The average difference between MRI A and MRI MS was 0.6 mm with LOA ranging from −0.88 mm to +2.10 mm (Figure 3). Again, the highest discrepancy was observed in Specimen 5, where MRI A showed the greatest variability compared to MRI MS, with a difference reaching 3.3 mm.

The MRI SA versus MRI MS comparison demonstrated a mean difference of 0.09 mm, with LOA from −0.28 mm to +0.48 mm. The agreement was more consistent compared to MRI A, suggesting that manual correction significantly improved segmentation accuracy.

The average difference between MRI SA and MRI A was 0.51 mm, with LOA from −0.73 mm to +1.75 mm. This indicated that while MRI SA reduced segmentation errors compared to MRI A, deviations remained in some specimens.

## DISCUSSION

The main finding of this study is that MRI‐based 3D knee reconstructions using semi‐automated segmentation with manual correction (MRI SA) significantly improved accuracy over fully automated segmentation (MRI A) and approached the performance of manual segmentation (MRI MS), which remained the most precise. While agreement of signed point‐to‐surface distances were found, the Bland‐Altman analysis demonstrated that MRI SA had a lower mean absolute point‐to‐surface distance compared to MRI A, reinforcing the importance of manual corrections in deep learning‐based segmentation workflows. These findings suggest that integrating AI‐assisted segmentation methods into clinical practice could speed‐up and facilitate 3D knee reconstruction for preoperative planning in knee ligament reconstructions, offering a radiation‐free alternative to CT‐based reconstructions.

Several studies have investigated the feasibility of MRI‐based 3D modelling for musculoskeletal applications. An open‐source, transformer‐based deep learning model, UNet‐R [5], was developed and trained for the automatic segmentation of knee anatomy. This model has demonstrated superior performance compared to other state‐of‐the‐art architectures (e.g., 3D U‐Net and V‐Net) in multi‐organ segmentation tasks involving structures of varying sizes [5]. Beyond preclinical studies demonstrating the feasibility of MRI for 3D osseous morphology assessment in animal models [10, 23], some authors have extended this research to human subjects. Moro‐Oka et al. reported that while MRI‐derived bone models were effective for kinematic shape‐matching, they displayed small but significant geometric deviations from CT‐based reconstructions, with RMS errors averaging 0.74 mm in sagittal translation and 1.48° in rotation [14]. More recently, Neubert et al. demonstrated that MRI‐based knee models achieved sub‐millimetre accuracy compared to CT, underscoring the potential of high‐resolution MRI sequences in precise 3D osseous modelling [17]. In 2020, Malloy et al. validated the accuracy of 1.5 T MRI‐derived femoral models, demonstrating their equivalence to CT and laser scan ground‐truth models [13]. However, the segmentation process relied on manual methods, making it time‐consuming due to the absence of deep learning automation. Despite this limitation, their findings confirmed that MRI‐based 3D reconstructions can serve as a viable alternative to CT‐derived models, offering radiation‐free imaging while preserving high anatomical fidelity.

The accuracy of deep learning‐based segmentation has been a growing area of research, with studies such as those by Kulseng et al. demonstrating that CNN‐based segmentation can achieve high levels of anatomical delineation but still requires manual correction for optimal accuracy [7]. Similarly, Niu et al. emphasised that integrating multi‐sequence MRI scans and advanced segmentation techniques could significantly improve soft tissue visualisation while maintaining high anatomical precision [18]. The improvements observed in our MRI SA versus MRI A agreement further validate these observations, confirming that automated segmentation remains effective and time efficiency but does not entirely eliminate the need for manual intervention.

To our knowledge, the current study is the only one to explore the feasibility of 3D knee reconstruction MRI based, using deep learning and comparing with laser scan. Also, our findings suggest that MRI‐based 3D knee reconstructions are feasible and highly accurate even at 1.5 T, supporting the broader implementation of MRI‐driven 3D modelling. Previous studies investigating MRI‐based 3D bone model generation have primarily utilised 3 T MRI [4, 17, 24, 25], which offers superior signal‐to‐noise ratios and enhanced spatial resolution. However, 3 T MRI is not universally accessible in routine clinical settings, particularly in institutions with limited resources or restricted access to high‐field imaging technology.

The validation of 1.5 T MRI‐based 3D knee reconstruction paves the way for its routine clinical use, particularly in ligamentous knee pathologies, where CT scans are not systematically performed. MRI is already the gold standard for assessing ligamentous and meniscal structures, providing excellent soft tissue resolution without exposing patients to ionising radiation. Integrating MRI‐based 3D reconstructions into clinical workflows could therefore enhance diagnostic accuracy and preoperative planning, optimising surgical decision‐making in these contexts.

Furthermore, replacing CT with MRI for 3D knee modelling eliminates radiation exposure, which is particularly relevant in young athletes undergoing multiple imaging sessions. This aligns with previous studies emphasising the importance of radiation‐free imaging techniques for musculoskeletal applications. Additionally, MRI‐based 3D models could play a role in preoperative simulations in order to improve long‐term surgical outcomes [8, 20, 22].

While this study validates the feasibility and accuracy of MRI‐based 3D knee reconstructions in a cadaveric setting, future research must focus on in vivo validation to confirm its clinical applicability. Conducting a similar study in patients with ACL‐deficient knees would provide valuable insights into the real‐world accuracy and usability of MRI‐derived 3D models. A direct comparison between MRI‐based 3D reconstructions and CT‐derived models in living subjects would help determine whether MRI can fully replace CT for preoperative planning, particularly in cases where radiation exposure should be minimised.

Beyond validation, the next step involves integrating MRI‐based 3D reconstructions into emerging surgical technologies, particularly augmented reality (AR) for arthroscopic procedures. Simultaneous Localisation and Mapping (SLAM) algorithms, which allow real‐time tracking and overlay of 3D models onto the patient's anatomy during surgery, could revolutionise arthroscopic knee surgery by improving spatial awareness and surgical precision [2, 27]. The combination of AI‐driven MRI segmentation, 3D modelling, and augmented reality guidance has the potential to enhance surgical precision while reducing operating time and long‐term outcomes.

### LIMITATIONS

Despite the promising results, several limitations must be acknowledged. One of the key observations in this study was that some fully automated (MRI A) segmentations produced outliers, requiring manual correction. This phenomenon was particularly evident in Specimens 5, 8, and 9, which exhibited point‐to‐surface distance discrepancies beyond the expected range. One possible explanation for this discrepancy is that the neural network used for segmentation was trained on MRI scans from living subjects, whereas the cadaveric knees used in this study exhibited different tissue signals, particularly in cartilage and soft tissue structures. Differences in tissue hydration, signal decay, and contrast between living and cadaveric tissues could have impacted the network's ability to accurately segment the anatomical structures, leading to inconsistencies that required manual correction.

Another limitation of this study is the small sample size (*n* = 11 knees), which may limit generalisability. Future studies should incorporate a larger cohort of specimens, including in vivo scans, to assess segmentation accuracy across a broader population. Additionally, while LS models served as the ground truth, minor surface artifacts from soft tissue remnants could have introduced variability in absolute point‐to‐surface distance calculations.

It is also important to note that manual correction remains a standard practice in 3D bone modelling. Several recent studies have emphasised that deep learning‐based segmentation, while powerful, still requires human validation and correction for clinical reliability [6, 26, 30]. Our findings align with this trend, reinforcing the idea that semi‐automated approaches combining AI‐driven segmentation with expert refinement are currently the most reliable method for generating high‐precision 3D knee models.

## CONCLUSION

This study confirms that MRI‐based 3D knee reconstructions using semi‐automated segmentation (MRI SA) provide a reliable and efficient alternative to manual segmentation (MRI MS), with superior accuracy compared to fully automated methods (MRI A). With a mean surface distance below 1.5 mm and an average processing time of 25 minutes, MRI SA demonstrates a favourable balance between precision and feasibility. The high agreement with laser scan ground‐truth models supports the adoption of MRI‐based 3D modelling in knee surgery planning as a radiation‐free, patient‐specific alternative to CT‐based reconstructions.

## AUTHOR CONTRIBUTIONS

Charles Pioger, Laura Marin and Yvon Gautier have made substantial contributions to conception, study design, acquisition/interpretation of data and in drafting the manuscript. Bertrand Sonnery‐Cottet, Pierre Imbert, Christian Lutz and Étienne Cavaignac have made substantial contributions to acquisition of data. Bertrand Sonnery‐Cottet, Yvon Gautier, Christian Lutz and Étienne Cavaignac have been involved in drafting or revising the manuscript critically. Each author has given final approval of the version to be published and agrees to be accountable for all aspects of the work in ensuring that questions related to the accuracy or integrity of any part of the work are appropriately investigated and resolved.

## CONFLICT OF INTEREST STATEMENT

Charles Pioger, Bertrand Sonnery‐Cottet, Christian Lutz and Étienne Cavaignac are consultants for Arthrex. Yvon Gautier, Julien Cléchet, Pierre Imbert, Christian Lutz, Étienne Cavaignac and Bertrand Sonnery‐Cottet have stock options in AREAS company. Laura Marin is a biomedical engineer in AREAS company. Yvon Gautier is the CEO of AREAS. The authors declare no conflicts of interest.

## ETHICS STATEMENT

Institutional review board approval (COS‐RGDS‐2022‐12‐025) was granted for this study which was conducted in strict accordance with the institutional ethical standards governing research involving human participants.

## Data Availability

Derived data supporting the findings of this study are available from the corresponding author on request.
